# Risk factors and leprosy incidence among contacts in Bangladesh: A multilevel analysis

**DOI:** 10.1371/journal.pntd.0013465

**Published:** 2025-09-05

**Authors:** Unnati Rani Saha, Abu Sufian Chowdhury, Johan Chandra Roy, Khorshed Alam, Daan Nieboer, Renate Verbiest-Richardus, Annemieke Geluk, Jan Hendrik Richardus

**Affiliations:** 1 Department of Public Health, Erasmus MC, University Medical Center Rotterdam, Rotterdam, The Netherlands; 2 The Leprosy Mission International, Nilphamari, Bangladesh; 3 Leiden University Center of Infectious Diseases, Leiden University Medical Center, Leiden, The Netherlands; Instituto Butantan, BRAZIL

## Abstract

**Background:**

The Maltalep trial in Bangladesh assessed whether single-dose rifampicin (SDR) given 8–12 weeks after bacillus Calmette–Guérin (BCG) vaccination was able to prevent excess leprosy cases due to BCG in contacts of newly diagnosed leprosy patients. After previous publication of the two years follow-up results of the trial, we now review the results after five years. Furthermore, to better understand the long-term protective effects of BCG against leprosy, we conduct post-hoc in-depth secondary statistical analyses based on the prospective interventional (randomized) Maltalep trial and a non-interventional (non-randomized) cohort study that was conducted simultaneously in the same project area.

**Methodology:**

The Maltalep trial is a single center, cluster-randomized controlled trial consisting of two arms. In one arm, SDR was given 8–12 weeks after BCG vaccination (SDR+), in the other arm no SDR was given after BCG revaccination (SDR-).

**Results:**

The Maltalep trial included 1,552 index patients. Of these, 14,986 eligible contacts were randomized into two arms SDR- and SDR+ of the trial. During the 5-year observation period, 95 and 100 new cases appeared among the contacts in two arms SDR- and SDR+ , respectively. Overall, there was no statistically significant difference in the leprosy incidence between the contacts of two arms of the trial. The non-intervention cohort included 554 index patients and 4,216 eligible contacts, with a total of 82 new leprosy cases appearing during the 5-year observation period. After adjustment for risk factors, the leprosy incidence was statistically significantly 1.70 [95% CI (1.03-2.80)] times higher in the contacts of the non-intervention cohort as compared to the contacts in the Maltalep trial. In the Maltalep trial, adjusted for both observed and unobserved differences, SDR- arm contacts of MB, slit skin smear (SSS) positive, blood-related (brother/sister, child, parent), and ‘blood-related other’ to index patients had higher risks for leprosy (AOR 2.35; 95% CI: 1.20-4.60; AOR: 6.35; CI: 2.42-16.72; AOR: 4.34; 95% CI: 1.83-10.26 and AOR: 3.07; 95% CI: 1.37-7.90) compared to PB, SSS negative, and not blood-related index patients. Household members of index patients had an increased risk (AOR: 2.60; 95% CI: 1.30-7.27) for leprosy. In the SDR+ arm, leprosy incidences were statistically significantly less in the contacts of MB, SSS positive, and ‘blood-related other’ index patients as compared to the same kind of contacts in the SDR- arm. Leprosy incidence increased with age of contacts, with a peak at age group 45+ years (AOR:3.45; 95% CI: 1.44-8.23).

**Conclusions and recommendations:**

BCG vaccination of contacts is effective in preventing leprosy, overall there is no clear benefit of adding SDR after BCG to reduce the number of excess leprosy cases after vaccination. SDR after BCG, however, appears effective to prevent leprosy in contacts of MB patients, smear positive index patients, and second degree blood-related contacts of index patients. Genetic relationship is a more profound risk factor for leprosy in contacts than being a household contact only. Leprosy incidence is clustered at levels of index patients and contacts, and this should be taken into account when assessing the effect of risk factors.

## Author summary

The Maltalep trial in Bangladesh assessed whether chemoprophylaxis with single-dose rifampicin (SDR) given after bacillus Calmette–Guérin (BCG) vaccination was able to prevent excess leprosy cases due to BCG in contacts of newly diagnosed leprosy patients. Simultaneously, a non-intervention cohort of leprosy patients was followed to establish new cases among their contacts. We now report the results after following these contacts over 5 years instead of 2 years as reported previously. There was no benefit of adding SDR after BCG vaccination to prevent leprosy, but SDR given after BCG prevented leprosy in contacts of MB index patients, smear positive index patients, and contacts of index patients who are blood-related in the second degree (e.g., cousins, etc.). Genetic relationship is a larger risk factor for leprosy in contacts than being a household contact only. After adjusting for risk factors, time variation in the enrolment of contacts and clustering effects, the number of new leprosy cases among contacts after 5 years was 70% higher in the non-intervention cohort as compared to the contacts of the trial where only BCG and both BCG + SDR were given. Leprosy incidence was clustered at levels of index patients and contacts, and this should be taken into account when assessing the effect of risk factors. The results of this study will inform leprosy control policy regarding the protective effects of BCG and SDR to stop transmission of *M. leprae*.

## Introduction

Leprosy is a neglected tropical disease (NTD) caused by *Mycobacterium leprae* or in few cases by *M. lepromatosis*. The pre-dominant mode of transmission of *M. leprae* is from person to person and thought to be through aerosols (inhalation) [1]. The observed annual trends in new case detection globally indicate a continuing transmission of *M. leprae* in many leprosy-endemic countries. Therefore, the World Health Organization (WHO) introduced chemoprophylaxis with single-dose rifampicin (SDR) as means for interrupting transmission and preventing new cases of leprosy [2]. This is part of the Global Leprosy Strategy 2021–2030 as part of the NTD road map 2021–2030 with the goal of eliminating leprosy (i.e., interruption of transmission) [3].

Bangladesh has the fifth highest number of leprosy cases in the world [4]. The northeast (Rangpur, Nilphamari, and Saidpur districts), southeast (Cox’s Bazar), and central Dhaka areas are known to be highly endemic for leprosy [5,6]. Around 4,000 new cases were detected annually in the years 2018, 2019, 2020, and 2023 respectively, and 2,872 and 2988 in 2021 and 2022, respectively, possibly reflecting the effect of the COVID pandemic [7].

SDR administered to close contacts of newly diagnosed leprosy patients reduces the risk clinical leprosy among these contacts considerably, in particular if the contacts had received childhood Bacillus Calmette-Guérin (BCG) vaccination [8,9]. BCG is a vaccine against tuberculosis (TB) and is routinely given to infants as part of the neonatal immunization scheme in many parts of the world. Moreover, BCG is also recognized as protecting against leprosy [10–13]. A meta-analysis by Setia *et al*. in 2010 showed an overall protective effect of BCG by 26% (95% CI 14–37%), and 61% (95% CI 51–70%) in experimental and observational studies, respectively [12]. In leprosy contacts, BCG given at infancy and SDR given during contact tracing protected against leprosy by 57% [95% CI: 24–75%] and 58% [95% CI: 30–74%], respectively. The combined strategies showed a protective effect of 80% (95% CI: 50–92%) [9]. Brazil has officially recommended BCG since the early 1970s for household contacts of leprosy cases as a booster to routine neonatal BCG vaccination. Since 1991, the Brazilian Ministry of Health has advised two doses of BCG to be administered to these leprosy household contacts. In 2008 Duppre *et al.* showed a 56% protection by the BCG booster, but the risk of tuberculoid leprosy among the contacts during the initial months after the BCG booster appeared high [14].

To substantiate the increased risk of paucibacillary (PB) leprosy after BCG, the Maltalep trial was initiated in Bangladesh in 2012 with the primary aim to determine whether possible excess cases in the first year after immunoprophylaxis with BCG could be prevented by chemoprophylaxis with SDR [15]. Firstly, it was shown that indeed a high proportion of healthy contacts of leprosy patients presented with PB leprosy within 12 weeks after receiving BCG vaccination, possibly as a result of vaccine-boosted cell-mediated immunity by homologues of *M. leprae* antigens in BCG, which originates from the related *Mycobacterium bovis* [11]. Secondly, it was shown that in the first year that SDR was given after BCG vaccination, the incidence of PB leprosy among contacts was reduced by 42%. This, however, was a statistically non-significant reduction due to the limited number of new leprosy cases after SDR administration [16]. Simultaneously with the Maltalep trial, a (non-randomized) non-intervention cohort of new leprosy patients was followed in the same project area to establish incident cases among their contacts during follow-up.

Through the data of the Maltalep intervention trial and non-intervention cohort over a 5-year period, we aim to 1) establish (characteristics of) new leprosy cases among contacts, and 2) perform post-hoc in-depth secondary analysis of the relationship between leprosy incidence and associated risk factors. The comparison of risk factors and leprosy incidence of both cohorts may help to better understand the impact of the intervention.

In-depth analysis of incidence and associated risk factors requires data availability on an array of socio-demographic, lifestyle, environmental, and clinical risk factors. Analysis that cannot include all relevant covariates leads to unobserved (hidden) differences in leprosy incidence. Although, randomization in the trial was at individual (new patient) level, the contacts of new patients may differ in both their observed and unobserved characteristics over the two intervention arms. Through differences in observed and unobserved behavioral, biological, and environmental factors, transmission of *M. leprae* from one person to another may differ in the contact groups of the two arms of the Maltalep trial, and in the Maltalep trial and non-intervention cohort. The lack of correction for such unobserved differences will bias the observed covariate effects [17,18], and differences in observed characteristics raises the issue of selection bias. Existing studies on determinants of leprosy incidence rarely address these issues. Thus, we performed advanced statistical analysis to adjust for differences in observed and unobserved factors and randomness of the data to estimate the effects of potential risk factors of leprosy incidence.

## Methods

### Ethics statement

The Maltalep trial and Ideal study received ethical approval by The National Research Ethics Committee (Bangladesh Medical Research Council) under Ref. No. BMRC/NREC/210-2013//1534). For the current study, based on secondary data analysis, no additional ethical approval was needed. Formal written consent was obtained from the study participants.

### Trial registration

Netherlands Trial Register: NTR3087.

### Population

The study population is newly diagnosed leprosy patients and their contacts. Patients were enrolled in the study by the Rural Health Program (RHP) of The Leprosy Mission International Bangladesh (TLMI-B) located in Nilphamari district in northwest Bangladesh. The population is from the four districts of Rangpur, Nilphamari, Panchghar, and Thakurgaon with a population size of around 7 million [19]. In these districts approximately 800–900 new leprosy patients are detected each year. The population is mainly rural, but the area also includes six main towns.

### Study design

The Maltalep trial is a cluster randomized controlled trial, using the newly diagnosed leprosy patient (index patient) as unit of randomization, to determine the effectiveness of SDR after BCG vaccination to prevent excess leprosy in close contacts due to BCG [15]. Index patients were included who had been diagnosed with leprosy according to the RHP guidelines, which follow the National Leprosy Control Programme of Bangladesh. From 2012 to 2017, close contacts of new patients were surveyed and enrolled in the study. Of these contacts, 57% had a visible BCG scar, indicating previous BCG vaccination during childhood. During enrolment each eligible contact was given BCG vaccine. Contact groups of newly diagnosed leprosy patients were allocated randomly to one of the two study arms of the Maltalep trial.

The non-intervention cohort was part of immunological studies referred to as the IDEAL study [20]. This study was conducted simultaneously in the same RHP project area and following the same RHP guidelines. The study participants were recruited between 2013 and 2018 [15,20].

**Fig 1:**
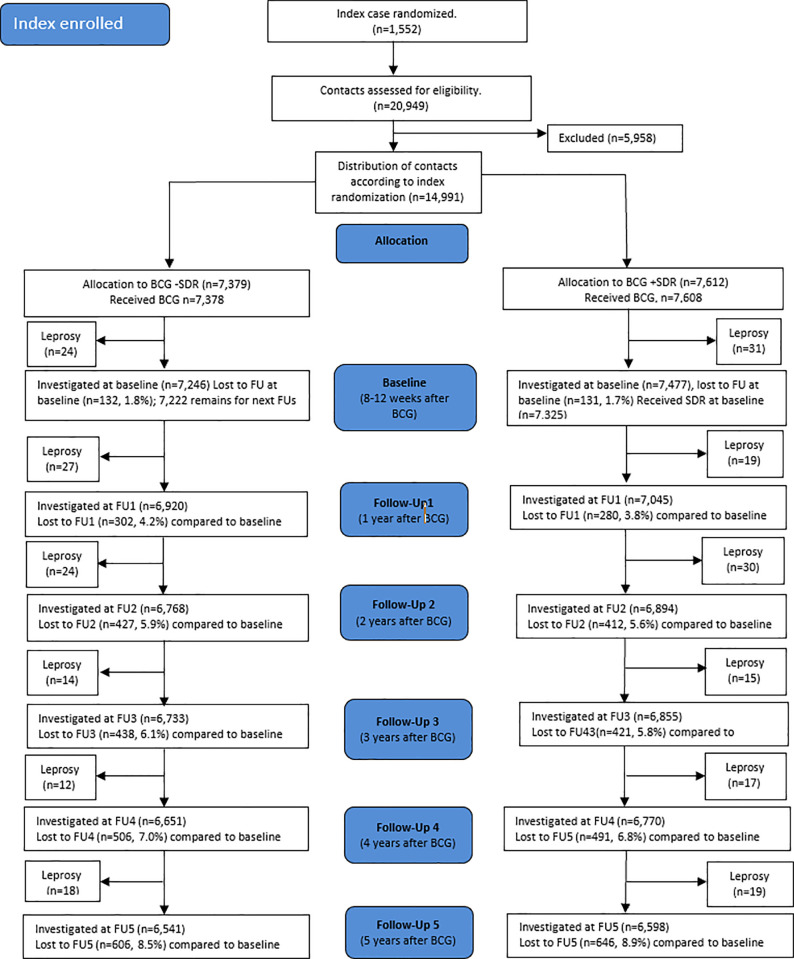
Maltalep trial: Flow chart of leprosy incidence and lost to follow-up.

SDR was administered to all contacts of the SDR+ arm of the trial 8–12 weeks after BCG vaccination at intake, which is the study time point referred to as baseline (Fig 1). During the intermediate period of 8–12 weeks before SDR, there were in total 55 new leprosy cases observed; 24 in the SDR- arm, and 31 in the SDR+ arm. Of these 55 new cases, 54 cases were PB (98%) [11]. These 55 cases are not taken into account in our analysis.

**Fig 2:**
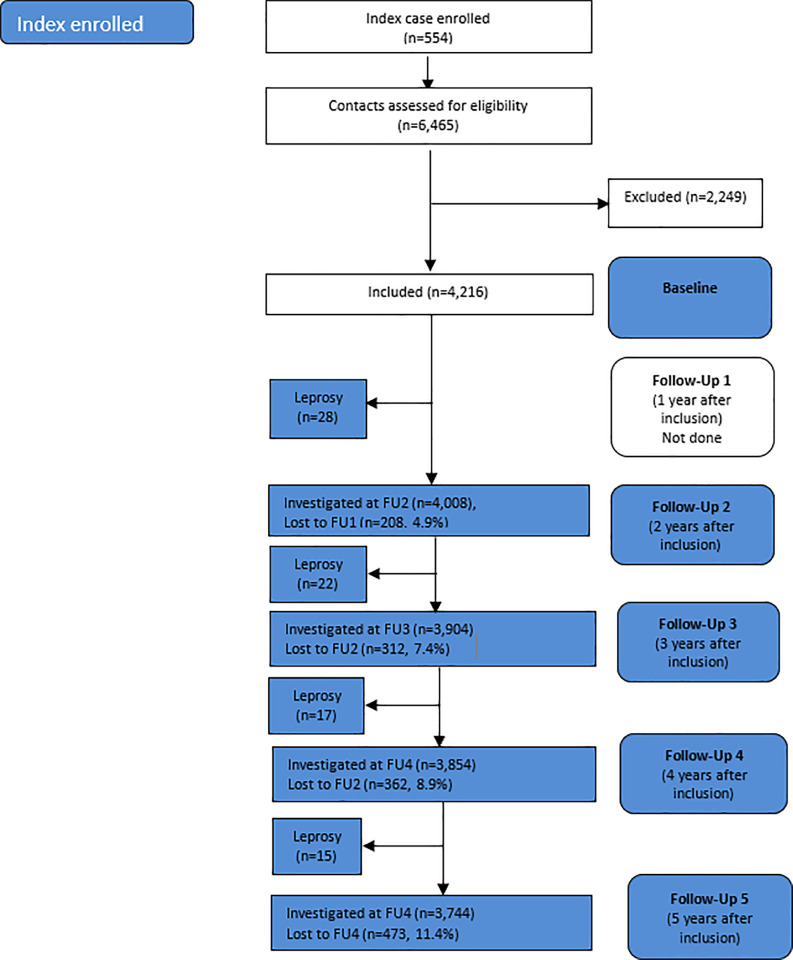
Non-intervention cohort: Flow chart of leprosy incidence and lost to follow-up.

Thereafter, the contacts in both arms were followed-up (FU) each year, referred to as FU1, FU2, FU3, FU4, and FU5, respectively. Contacts who, for instance, were not traceable at a FU, may appear again in a following FU. Thus, if a contact disappears (e.g., at FU1), but appears again at FU2, and is diagnosed as a leprosy case within 6 months of the date at FU1, the contact was then recorded for FU1. This procedure was followed for each follow-up period. The follow-up of the non-intervention cohort started two years after enrollment of an index case. This first follow-up after baseline is referred to as FU2 and then followed up yearly, referred to as FU3, FU4, and FU5 (Fig 2).

Overall, in the Maltalep trial, 2,986 of 14,986 (19.9%) contacts were lost to follow-up at any time during the 5 years, but 81 contacts (<1%) were lost to follow-up completely over the 5-year follow-up period. In the non-intervention cohort no contacts were lost to follow-up completely over the 5-year follow-up period, but 1,736 of 4,216 (41.2%) contacts were lost to follow-up at any FU time point within the five years.

## Data

### Data sources

Data came from the routine leprosy control data of the RHP, together with the data specifically collected for the Maltalep trial and the non-intervention cohort. RHP maintains a ‘Lepra’ data file that includes the routine data of all leprosy patients of the RHP over a period of more than 40 years. All datasets are maintained at the RHP site in Nilphamari. The database used for the present study is linked with all these three databases through ‘pseudonymized’ codes. All data procedures comply with data protection guidelines as required by Erasmus MC, University Medical Center Rotterdam in the Netherlands. As Erasmus MC researcher, the lead applicant is authorized to use the RHP data for analysis, ensuring data security procedures.

### Outcome measures

The primary outcome measure of the study is the incidence rate of leprosy per 10,000 population at risk among contacts during a 5-year observation period. Leprosy disease is classified as paucibacillary (PB) or multibacillary (MB) following the definition of the WHO [21].

The primary outcome measure of the Maltalep trial was the incidence rate of leprosy per 10,000 population at risk among contacts during a 2-year observation period [16]. For the current study we have extended the observation period to 5 years. This analysis, including data from the separate non-intervention cohort, represents additional secondary analysis not described in the original Maltalep study protocol.

### Covariates

An array of clinical and socio-demographic information was recorded in both studies. We included the following covariates in our statistical analysis: gender (male and female), age in years (age groups 5–14, 15–29, 30–44 and 45+ years), leprosy classification (MB and PB), value of a SSS for the presence of *M. leprae* in new leprosy patients (0–6), and location (district). Contacts were categorized according to their genetic and physical distance to the index patients [16,22]. Genetic distance was categorized as ‘*blood-related*’ (brother/sister, child, parent), ‘*blood-related other*’ (e.g., cousin, grandchild, etc.), and ‘*not blood-related or unclear*’ (all others) [22]. Physical distance was categorized as *‘sharing the same roof and kitchen*’ (referred to as ‘household member’) versus ‘*not a household member*’. To identify the two arms of the trial and the non-intervention cohort, we created a categorical variable with values (1) ‘BCG’, (2) ‘BCG+SDR’, and (3) ‘non-intervention’, respectively. This variable will be treated as intervention arms ‘BCG’ and ‘BCG+SDR’ *vs.* ‘non-intervention’ cohort.

### Data management

We used STATA version 15 for the data cleaning, coding, and statistical analysis. Data were recorded using the relational database software ACCESS. We used a unique ID to combine socio-demographic and clinical variables in one dataset, and missing observations checked, and discrepancies corrected. For example, the variable SSS-positive and SSS-negative cases were 7.58% and 86.05%, respectively. The remaining 6.37% cases were refusals or missing. The percentage distribution of leprosy in the combined refusal and missing cases was similar as the percentage distribution of leprosy of those cases who had a negative SSS value. Thus, we recoded these missing and refusal cases into negative SSS value.

### Descriptive analysis

Descriptive statistical analysis was applied to compute leprosy incidence rates with 95% confidence intervals (CI) of the two arms of the Maltalep trial and the non-intervention cohort over a five-year follow-up period, and to describe the distribution of the covariates over study groups, and the distribution of the primary outcome measure (leprosy) over study groups and covariates, respectively. The Pearson chi-square statistic test was used in bivariate statistical analysis to examine associations between each variable and the primary outcome measure. Bivariate results are presented with 95% confidence intervals and p-values.

### Risk factor analysis

Firstly, we performed inverse propensity score weighting (IPW) analysis [23,24] to balance the two groups (SDR+ and SDR-) of the Maltalep trial by giving each data point a different weight so that the weighted distribution of data in each group is similar. This addresses selection bias and differences in characteristics of socio-demographic and clinical indicators that we observed at baseline.

Secondly, evidence suggests that leprosy is clustered in some locations and in some populations with poor socioeconomic conditions [5,25,26]. Therefore, we assumed that the incidence of leprosy is not random but clustered at levels of locations and of individuals like newly diagnosed leprosy patients and contacts. Studies often cannot include all potential risk factors in the analysis, which remain as hidden (or unobserved). For example, in this study we could not control for factors like lifestyle, environmental risks, and innate biological weakness. Therefore, such factors remain as hidden differences, called ‘frailty’, in the incidence of leprosy. The variability between new patients and between contacts within a cluster of a new patients leads to different ‘shared frailty’ for the groups. The term ‘shared frailty’ expresses that individuals in a cluster share the same frailty, which is constant over time. Controlling for this shared frailty in the statistical analysis influences the observed covariate effects [17]. Furthermore, due to similarity of the observations within clusters, each individual within a cluster provides less information compared to an individual in a non-clustered trial.(28) Therefore, clustered designs require larger sample sizes compared to non-clustered randomized designs, and special statistical analyses that account for the fact that observations within clusters are correlated [27].

Thus, using inverse propensity score weight, we performed covariates adjusted two-level (multilevel) mixed effects logistic regression models to examine the cluster adjusted risk factors in the odds of leprosy outcome in the contacts of the two arms of the Maltalep trial. Multilevel analysis considers the variation in leprosy outcome and splits this variation into the part that is attributable to observed risk factor differences (e.g., clinical, demographic, or behavioral characteristics), and the part that is attributable to unobserved differences (unobserved factors). The unobserved differences split into (1) the part attributable to differences between contacts of new patients and (2) the part attributable to differences between new patient context (contextual effects). It is possible that many observed differences in risk factors for disease can be explained by the unobserved differences like genetic and/or shared environmental context in which individuals interact. Thus, it is important to consider how these unobserved factors might influence the risk of leprosy outcomes for individuals in the contact groups, particularly given that some contacts are genetically related to new patients. To address these unobserved differences, we used a multilevel modelling approach and fit a random intercepts model for contact groups and new patients, adjusted for observed risk factors. Concerning the causal validity, random effects are assumed to be uncorrelated with the error term and with the covariates. Since covariates were measured at one time-point (cross sectional) it is most likely that assumptions hold.

We determined the statistical significance of the covariate effects (fixed effects) by odds ratios (OR) with 95% confidence intervals (CIs). The p-value of <0.05 indicates statistical significance. We used the intra-cluster correlation coefficient (ICC) to measure the variations between new patients and between contacts within same new patient contact group. Leprosy also may cluster within geographic areas. To capture geographic variations, we adjusted for four district (higher levels of geographic regions) dummies as covariates in the multilevel analysis. Also, we attempted to adjust for Upazilla level clustering (lower levels of geographic regions) in multilevel analysis. However, we did not find clustering effects and no changes in other covariates effects of the multilevel results. Thus, for the convenience of the analysis we performed two-level (new patients and contacts within a new patient) multi-level analysis.

We tested the model fits comparing the loglikelihood value, the Akaike information criterion (AIC), and the Bayesian information criterion (BIC). The model with the lowest information value was chosen as the benchmark model (multilevel mixed-effects model with interaction terms; Model 4). We performed the calculation of covariates effects for the SDR+ arm by multiplying with the significant interaction effects of the SDR- arm and with interaction effects of the SDR+ arm, for example, to show difference in terms of odds ratios between PB and MB index patients of SDR+ arm, (2.35 x 0.39, which is 0.91; Model 4), between SSS positive and negative index patients (6.36x0.18, which is 1.14; S10 Table) and between ‘blood-relation other’ and ‘not blood-relation’ index patients (3.07 x 0.30, which is 0.92; Model 4).

Furthermore, by means of covariate adjusted multilevel mixed-effects binary logistic regression models, we estimated the coefficients of association between leprosy outcome and potential risk factors combining both the Maltalep trial and non-intervention cohort data. We examined the odds of leprosy in the contacts of non-intervention cohort against the contacts in the Maltalep trial. We report the significant (p < 0.05) parameter estimates obtained from the descriptive and regression models in the results section of the paper.

## Results

### Leprosy incidence over five-year study period

The Maltalep trial included 1,552 newly diagnosed leprosy (index) patients and 14,991 eligible contacts for BCG vaccination. We excluded 5 of these contacts (1 from the SDR- and 4 from the SDR+ group) because in fact they had not received BCG. The remaining 14,986 eligible contacts were distributed into two groups: BCG with SDR (SDR+) and BCG without SDR (SDR-) (Fig 1). In the pre-baseline period of the trial, the time between BCG vaccination and the provision of SDR, 24 and 31 leprosy cases were recorded in the SDR- and SDR+ arms, respectively. During the five-year observation period, 195 leprosy cases were recorded, 95 and 100 in the SDR- and SDR+ arms, respectively.

The non-intervention cohort included 554 newly diagnosed leprosy (index) patients and 4,216 eligible contacts, representing the baseline for this cohort (Fig 2). A total of 82 confirmed leprosy cases were observed over a five-year period.

**Fig 3:**
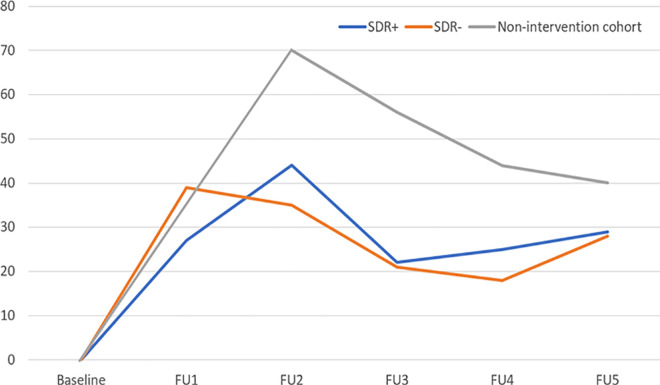
Leprosy incidence per 10,000 population.

Table 1 and Fig 3 show the incidence rates per 10,000 population at risk. Taking both MB and PB leprosy together, the incidence rates of leprosy at one year (FU1) are 39 [95% CI:24–54] and 27 [95% CI: 15–39] in the SDR- and SDR+ arms, respectively. The reduction in incidence of leprosy in the SDR+ group in year 1 is 30%. At FU2, the incidence rate is 44 [95% CI: 28–59] in the SDR + arm compared to 35 [95% CI: 21–50] in the SDR- arm. These differences are statistically not significant, nor are the differences between the arms in the subsequent FU’s. The leprosy incidence in the non-intervention group was higher across all follow-up points compared to both arms of the Maltalep trial.

**Table 1:** Leprosy incidence per 10,000 Population in contacts of new leprosy patients: Maltalep trial (N = 14,547) and non-intervention cohort (N = 4,216).

Year Follow-up	Maltalep Trial, SDR- arm				Maltalep Trial, SDR + arm				Non-intervention cohort			
	Leprosy	Incidence rate per 10,000 Population	Number at risk	Loss to FU	leprosy	Incidence rate per 10,000 Population	Number at risk	Loss to FU	leprosy	Incidence rate per 10,000 Population	Number at risk	Loss to FU
Baseline	–	–	7,222	–	–	–	7,325	–	–	–	4,216	–
1 year FU 1	27	39 [95% CI: 24–54]	6,920	4.2% (n = 302)	19	27 [95% CI: 15–39]	7,045	3.8% (n = 280)	–	Not Done	–	–
2-year FU 2	24	35 [95% CI: 21–50]	6,768	5.9% (n = 427)	30	44 [95% CI: 28–59]	6,894	5.6% (n = 412)	28	70 [95% CI:47–102]	4,008	4.9% (n = 208)
3-year FU 3	14	21 [95% CI: 10–32]	6,733	6.1% (n = 438)	15	22 [95% CI:11–33]	6,855	5.8% (n = 421)	22	56 [95% CI: 32–79]	3,904	7.4% (n = 312)
4-year FU 4	12	18 [95% CI: 8–28]	6,651	7.0% (n = 506)	17	25 [95% CI: 13–37]	6,770	6.8% (n = 491)	17	44 [95% CI: 23–65]	3,854	8.9% (n = 362)
5-year FU 5	18	28 [95% CI: 15–40]	6,541	8.5% (n = 606)	19	29 [95% CI: 16–42]	6,598	8.9% (n = 646)	15	40 [95% CI: 20–60]	3,744	11.4% (n = 473)
**Total leprosy (FU1–5)**	95				100				82			

Note: - CI, confidence interval; FU, Follow-up. - Numbers at risks for FU1, FU2, FU3, FU4, FU5 and loss to follow-ups are given as per Figs 1 and 2

The incidence rates per 10,000 population at risk in the non-intervention cohort are 70 [95% CI:47–102], 56 [95% CI: 33–80], 44 [95% CI: 23–65], and 40 [95% CI: 20–60] at two years (FU2) following each one year at FU3, FU4, and FU5, respectively. The leprosy incidences in contacts over a five-year period FUs are higher in the non-intervention cohort compared to the Maltalep trial. The overall five-year incidence rates for the SDR-, SDR+ and non-intervention cohort are 137 [95% CI: 110, 163], 132 [95% CI: 105, 158] and 195 [95% CI: 153, 236], respectively. The incidence rate in the non-intervention cohort differ significantly (p = 0.00) with the incidence rate in the two arms of the Maltalep trial taken together.

Chi-squared tests show no statistically significant difference at the p = 0.05 level in the incidence rates between the SDR- and SDR+ arms of the Maltalep trial at any of the observation periods (S1 Table). The likelihood of leprosy is significantly higher at FU2 (p < 0.05), FU3 (p < 0.001), and FU4 (p < 0.05) in the contacts of the non-intervention cohort compared to the incidence rates in the contacts of the Maltalep trial.

S2 and S3 Tables show leprosy in contacts by PB/MB status. The absolute number of PB leprosy after 5 years FU is 85, 84, and 72 in the SDR-, SDR+ arms, and non-intervention cohort, respectively (S2 Table). The difference in PB status between the two intervention groups on the one hand, and the non-intervention cohort on the other hand over 5 years is statistically significant (p = 0.00). There is a marked reduction in PB cases in the SDR+ group at FU1 (14 *vs.* 23; 40%) compared to the SDR- group, but this is statistically not significant at the p = 0.05 level. The absolute number of MB leprosy after 5 years follow-up is 10, 16, and 10 in SDR-, SDR+ , and non-intervention cohort, respectively (S3 Table). These differences are statistically not significant at the p = 0.05 level.

Additionally, S4–S7 Tables report the (unadjusted) risk factors at each follow-up over the groups. Leprosy incidence is significantly lower [AOR: 0.33; 95% CI:0.11,1.01; p = 0.05] in the contacts of MB index patients at FU2 in the SDR+ group as compared to the contacts of MB index patients in the SDR- group. Alternatively, leprosy incidence was significantly higher in contacts of SDR+ group as compared to the PB contacts in the SDR- group. Leprosy incidences are significantly higher at FU4 in the contacts who are not member of household member of index patients [AOR: 2.39; 95% CI: 1.27, 4.49; p = 0.00] and at FU5 in the 5–14 years old contacts group [AOR: 5.56; 95% CI: 1.49, 20.73; p = 0.01] in the non-intervention cohort as compared to the contacts of Maltalep trial.

### Baseline contact characteristics and leprosy incidence

Table 2 gives the distribution characteristics (covariates) of contacts of index patients at the time of intake. This table provides the basic information for further risk factor analysis.

**Table 2:** Characteristics at intake of contacts of index leprosy patients (Maltalep trial, N = 14,986) by treatment allocation, and the non-intervention cohort (N = 4,216).

Variable	Maltalep trial					Non-intervention cohort		
	SDR- N = 7,378	Valid %	SDR + N = 7,608	Valid %	p-value*	N = 4,216	Valid %	p-value**
**Age of contacts (years) a**					0.94			0.00
5-14	2,200	29.83	2,300	30.23		998	23.67	
15-29	2,051	27.81	2,113	27.77		1298	30.79	
30-44	1,585	21.49	1,611	21.18		934	22.15	
45+	1,540	20.88	1,584	20.82		986	23.39	
**Gender of contacts**					0.36			0.34
Male	3,358	45.51	3,406	44.77		1938	45.97	
Female	4,020	54.49	4,202	55.23		2278	54.03	
**Genetic distance to index patients**					0.03			0.00
Blood-related (brother/sister/child/parent)	1,662	22.53	1,647	21.65		1131	26.83	
Blood-related (other)	2,176	29.49	2,391	31.43		1197	28.39	
Not blood-related (or unknown)	3,540	47.98	3,570	46.92		1888	44.78	
**Physical distance to index patients**					0.01			0.00
Household member (sharing roof and kitchen)	1,071	14.52	998	13.12		665	15.77	
Not a household member	6,307	85.48	6,610	86.88		3551	84.23	
**Leprosy classification index patient**					0.00			0.00
PB	5,008	67.88	5,364	70.50		2548	60.44	
MB	2,370	32.12	2,244	29.50		1668	39.56	
**Slit skin smear result index patient**					0.00			0.00
Present (value 1–6)	597	8.09	539	7.08		5183	12.29	
Absent (*value 0)*	6,267	84.94	6,628	87.12		3,533	83.80	
Refuse	273	3.70	243	3.19		165	3.91	
Missing	241	3.27	198	2.60		0	0	
**Occupation index patient**					0.00			0.00
Daily labour	775	10.50	607	7.98		642	15.45	
Others	6,603	89.50	7001	92.02		3,514	84.55	

* Significant levels of difference between two arms SDR- & SDR+ ; ** significant levels of difference between the two studies Maltalep trial and the non-intervention cohort;

**a** 2 cases were missing for age of the contacts; BCG scar variable was excluded because this information was not available in the non-intervention cohort.

In both studies a total of 19,202 contacts (14,986 from the Maltalep trial and 4,216 from the non-intervention cohort) were available for the analysis. The p-values indicate the level of statistically significant differences in the distribution of covariates between 1) the two arms SDR- and SDR+ of the Maltalep trial (column five of Table 2), and 2) between the Maltalep trial on the one hand, and the non-intervention cohort on the other (last column of Table 2).

Apart from age, gender, and BCG scar present/absence, the most statistically significant differences between the two arms are for the variables genetic and physical distance, PB/MB status, and SSS score in index patients. The percentage of contacts as household member of index patient (14.5% *vs.* 13.1%), of MB index patients (32.1% vs 29.5%), of SSS positive (≥1) index patients (8.1 *vs.* 7.1), and of index patient with occupation ‘labor’ (10.5 *vs.* 8.0) is higher in the SDR- group compared to the SDR+ group. The percentage of contacts being ‘blood-related other’ is higher in the SDR+ group (31.4% vs. 29.5%). Although index cases and their contacts were randomly selected into the two arms of the Maltalep trial,

Table 2 shows clearly that randomization is not perfect regarding the distribution of the variables involved. This underscores the rational of conducting inverse propensity score weight (IPW) analysis and adjusting for this in the multilevel analysis.

Furthermore, in the comparison between the Maltalep trial groups and the non-intervention cohort, apart from gender, the characteristics of contacts vary statistically significantly between the SDR+ group and the non-intervention cohort. The largest difference is observed for the percentage of MB index patient between non-intervention cohort and SDR+ groups (39.6% *vs.* 29.5%) at intake.

### Bi-variate associations of risk factors and leprosy incidence

Before multivariable multilevel logistic analysis, we performed bi-variate analysis of risk factors and leprosy incidence. Table 3 shows the results, giving the unadjusted risk factors associated with the odds of leprosy incidence.

**Table 3:** Cluster unadjusted bi-variate association of risk factors and leprosy incidence.

Variables	Maltalep trial (n = 14,986)			Combined two studies Maltalep and non-intervention trial (n = 19,202)		
	Odds ratios	CIs	p-values	Odds ratios	CIs	p-values
**Intervention arm**						
SDR-	1			1		
SDR+	1.02	0.77–1.35	0.88			
Non-intervention cohort	n.a.	n.a.	n.a.	1.47	1.14–1.91	0.00
**Age of contacts (years)**						
5-14	1			1		
15-29	1.37	0.91–2.07	0.13	1.12	0.79–1.58	0.52
30-44	1.78	1.18–2.68	0.01	1.63	1.16–2.30	0.01
44+	1.84	1.22–2.78	0.00	1.65	1.18–2.32	0.00
**Genetic distance to index patients**						
Not blood-related (or unknown)	1			1		
Blood-related (brother/sister/child/parent)	2.52	1.81–3.51	0.00	2.10	1.59–2.76	0.00
Blood-related (other)	1.26	0.88–1.81	0.21	1.09	0.81–1.48	0.57
**Physical distance to index patients**						
Not a household member	1			1		
Household member (sharing roof and kitchen)	1.74	1.23–2.45	0.00	1.72	1.29–2.30	0.00
**Leprosy classification index patient**						
PB	1			1		
MB	1.38	1.03–1.84	0.03	1.38	1.08–1.75	0.01
**Slit skin smear result index patient**						
Absent (*value 0)*	1			1		
Present (value 1–6)	2.44	1.66–3.57	0.00	2.52	1.87–3.38	0.00
**Occupation index patient**						
Others	1			1		
Daily labour	1.39	0.90–2.131	0.14	1.45	1.03–2.03	0.03

n.a. indicates ‘not applicable’; BCG scar of index patient was not available in the dataset of non-intervention cohort.

Of 14,986 contacts, in total 195 leprosy cases occurred in the two arms of the Maltalep trial after SDR. We excluded contacts of both SDR+ and SDR- arms who developed leprosy after BCG vaccination but before SDR from the analysis. Contacts associated with MB index patients (OR: 1.38; 95% CI: 1.03-1.84; p < 0.05), SSS positive (≥1) index patients (OR: 2.44; 95% CI: 1.66-3.57; p < 0.001), blood-relation to index patient, i.e., brother/sister, child, parent (OR:2.52; 95% CI: 1.81-3.51; p < 0.001), and being a household member of index patient (OR:1.74; 95% CI:1.23-2.45) all had a statistically significantly higher chance of acquiring leprosy as compared with those not having these characteristics. Furthermore, there is an increased probability of leprosy incidence with increasing age of contacts, peaking at the age group 44+ (OR: 1.84; 95% CI: 1.22-2.78; p = 0.004).

In the non-intervention cohort, 82 contacts developed leprosy. We analysed a total of 19,202 contacts from both studies (Maltalep trial and non-intervention cohort), in which 277 (195 + 82) leprosy cases were observed. The odds of leprosy is 1.47 times higher in the contacts of the non-intervention cohort compared to the contacts of the Maltalep trial (CI: 1.14-1.91; p = 0.004). Contacts having a blood-relationship (OR: 2.10; CI: 1.59-2.76; p < 0.001), being a household member (OR: 1.72; CI: 1.29-2.30; p < 0.001), associated with MB index patient (OR:1.38; CI: 1.08-1.75; p < 0.05), contacts with higher age group 44+ (OR: 1.65; CI: 1.18-2.32; p = 0.004), being contact of male index patients (OR: 1.35; CI: 1.05-1.72; p < 0.05), and being associated with index patients’ occupation as ‘labor’ (OR: 1.45; CI: 1.03-2.03; p < 0.05) had statistically significantly higher odds of leprosy occurrence, respectively.

### Comparisons of models and adjusted risk factors: Maltalep trial

Table 4 presents the adjusted risk factors obtained from the multivariable and multilevel multivariable logistic regression models. The sample size included all eligible contacts of the Maltalep trial. Contacts who developed leprosy after BCG vaccination but before SDR was given (pre-baseline period), were excluded from the analysis. Model 1 shows the risk factors effects assuming no difference in risk factors effects over the SDR- and SDR+ arms. Model 2 shows risk factors effects assuming that effects are different over the two arms. This was done by including both main and interaction effects simultaneously in the model. The results of Model 1 and Model 2 are obtained from multivariable logistic regression analyses. These models do not adjust for the clustering effects. Model 3 shows risk factor effects obtained from multilevel mixed effects model whereas Model 4 of multilevel mixed effects shows that risk factors effects affect leprosy differently in contacts of new patients over the two arms. These models do adjust for the clustering effects. All four models were adjusted for the IPW weight. A sensitivity check of the use of IPW found that IPW it does not change the effects of risk factors (S8 Table). However, use of IPW helps to increase speed and convergence of the hierarchic nested nature multilevel mixed effects model. We consider Model 4 as our benchmark model (lowest loglikelihood, AIC and BIC values given at the bottom of Table 4). This model indicated that 41% of unobserved variation was explained by the differences between index patients and 60% by the differences between contacts within same index patient.

**Table 4:** Results of multivariable and multilevel mixed-effects logistic regression analysis, Maltalep trial after SDR was given, N = 14,547.

Variables	Model 1		Model 2		Model 3		Model 4	p-value
**Intervention**	AORs	p-value	AORs	p-value	AORs	p-value	AORs	
SDR-	1				1		1	
SDR+	1.07 (0.81–1.42)	0.63	1.01 (0.31–3.28)	0.99	1.15 (0.76–0.75)	0.52	1.35 (0.27– 6.76)	0.72
**Age of contacts**								
5-14	1				1		1	
15-29	1.69 (1.10–2.59)	0.02	1.84 (1.03– 3.28)	0.04	1.96 (1.09–3.51)	0.03	2.37 (1.04–5.38)	0.04
30-44	2.22 (1.45–3.38)	0.00	1.74 (0.93–3.25)	0.08	2.89 (1.52–5.49)	0.00	2.19 (0.90–5.31)	0.08
45+	2.19 (1.43–3.36)	0.00	2.47 (1.39–4.40	0.00	2.84 (1.47–5.46)	0.00	3.45 (1.44–8.23)	0.01
**Leprosy classification of index patients**								
Paucibacillary (PB1–5)	1				1		1	
Multibacillary (MB)	1.34 (0.99–1.82)	0.06	1.81 (1.16–2.81)	0.01	1.46 (0.91–2.34)	0.11	2.35 (1.20–4.60)	0.01
**Genetic distance to index patients**								
Not blood-related	1		1		1		1	
Blood-related (Brother/sister, child, parent)	2.34 (1.64–3.33)	0.00	2.91 (1.72–4.91)	0.00	3.21 (1.76–5.85)	0.00	4.34 (1.83–10.26)	0.00
Blood-related other	1.52 (1.04–2.21)	0.03	2.38 (1.39–4.06)	0.00	1.66 (0.99–2.79)	0.06	3.07 (1.30–7.27)	0.01
**Physical distance to index patients**								
Not a household member	1		1		1		1	
Household member (KR)	1.57 (1.07–2.31)	0.02	1.94 (1.15–3.27)	0.01	1.80 (1.04–3.11)	0.04	2.60 (1.23–5.51)	0.01
**Leprosy suspects at contacts enrolment**								
No	1		1		1		1	
Yes	1.74 (1.18–2.57)	0.01	1.74 (1.17–2.57)	0.01	1.88 (1.01–3.50)	0.05	1.92 (1.02–3.62)	0.04
**Time of contacts enrolment (year)**								
2012	1		1		1		1	
2013	0.59 (0.35–1.00)	0.05	0.58 (0.34–0.99)	0.04	0.51 (0.21–1.24)	0.14	0.50 (0.20–1.23)	0.13
2014	0.46 (0.27–0.80)	0.01	0.46 (0.27–0.80)	0.01	0.39 (0.16–0.99)	0.05	0.39 (0.15–1.00)	0.05
2015	0.66 (0.39–1.13)	0.13	0.67 (0.39–1.12)	0.12	0.58 (0.24–1.42)	0.23	0.57 (0.23–1.42)	0.22
2016	0.53 (0.30–0.93)	0.03	0.53 (0.30–0.93)	0.03	0.42 (0.16–1.10)	0.08	0.43 (0.16–1.13)	0.09
2017	0.42 (0.14–1.22)	0.11	0.41 (0.14–1.21)	0.11	0.27 (0.05–1.34)	0.11	0.26 (0.05–1.33)	0.11
**Interaction with SDR+**								
Multibacillary	–		0.52 (0.28-0.96)	0.04	–		0.39 (0.16-0.98)	0.05
Genetic distance (blood-related other)	–		0.43 (0.20-0.93)	0.03	–		0.30 (0.09-0.98)	0.05
**Sample size a**	14,542		14,542		14,542		14,542	
**Used IPW**	yes		yes		yes		yes	
**Loglikelihood**	-997.74		-989.46		-981.89		-974.02	
**Parameter**	19		29		21		31	
**AIC**	2033.46		2036.92		2005.78		2010.04	
**BIC**	2177.50		2256.74		2164.96		2245.02	
**ICC between index patients**	–		–		0.40		0.41	
**ICC between contacts within same index patient**	–		–		0.58		0.60	

**Note:** ICC Intra-cluster correlation; IPW Inverse probability weight. AIC Akaike Information Criteria; BIC Bayesian information criterion; adjusted risk factors for age of index patients, gender of both contacts and index patients, occupation of index patients as labor and interaction terms.

There was no statistically significant difference in the overall 5-years incidence of leprosy between the contacts in the SDR+ and SDR- arms of the Maltalep trial. Comparison of Model 1 and 2, and Model 3 and 4, showed that the statistically insignificantly higher leprosy incidence in the contacts of SDR+ arm was sensitive to risk factor sub-groups. We explained the results of Model 4 as benchmark results.

In the SDR- arm, the contacts of MB index patients had a statistically significantly higher risk of leprosy (adjusted odds ratio (AOR) 2.35; 95% CI: 1.20-4.60; p = 0.01) compared to the contacts of PB index patients. The risk of leprosy incidence was statistically significantly higher for blood-related (brother/sister, child, parent) and blood-related other contacts of index patients (AOR: 4.34; 95% CI: 1.83-10.26; p = 0.00 and AOR: 3.07; 95% CI: 1.30-7.27; p = 0.01) compared to contacts who were not blood-related to index patients. Household members of index patients had an increased risk (AOR: 2.60; 95% CI: 1.23-5.51; p = 0.01) to develop leprosy. Finally, leprosy incidence increased with age of contacts, with a peak at age group 45+ years (AOR:3.45; CI: 1.44-8.23; p = 0.01). Suspect leprosy cases identified in the contacts group during enrollment had an increased risk (AOR: 1.92; CI: 1.02-3.62; p = 0.04) to develop leprosy. Other factors in the model were not statistically significant.

In the SDR+ arm, using interaction terms between intervention and different covariates we found that 1) the contacts of MB index patients showed statistically significantly less risk to developing leprosy as compared to the contacts to MB index patients in the SDR- arm (AOR: 0.39; CI: 0.16-0.98; p = 0.05); 2) the contacts that are ‘blood-related other’ to index patients showed statistically significantly less risk (AOR: 0.30; CI: 0.09-0.98; p = 0.05) as compared to the contacts that are ‘blood-related other’ to index patients for SDR- arm; and 3) male contacts in the SDR+ arm appeared more likely to acquire leprosy (AOR: 2.04; 95% CI: 0.88- 4.71; p = 0.10) than the male contacts in the SDR- arm, but the difference is not statistically significant.

Multiplication with the significant interaction effects and with effects of the SDR- arm showed that for the contacts of the SDR+ arm, there was hardly any difference in leprosy incidence between PB and MB index patients, and between ‘blood-relation other’ and ‘not blood-related’ index patients. We also performed logistic regression analysis involving only contacts of the SDR+ arm and also found no differences between neither PB and MB index patients, nor ‘blood-relation other’ and ‘not a blood-relation’ index patients (S9 Table).

In an alternative model (S10 Table) with SSS values instead of PB/MB status of index patients, a profound association was found (AOR: 6.36; CI: 2.42-16.72; p = 0.00) for SSS positivity in index patients and leprosy incidence in all contacts of the Maltalep trial. As for MB index patients, contacts in the SDR+ arm of SSS positive index patients were statistically significantly less likely (AOR:0.18; CI: 0.04-0.75) to develop leprosy.

### Comparisons of adjusted risk factors: Maltalep trial and non-intervention cohort

We performed multilevel analysis for the combined dataset (Maltalep trial and non-intervention cohort). Risk factor adjusted logistic regression of the Maltalep trial and non-intervention cohort (Table 5) showed that contacts of the non-intervention cohort were statistically significantly more likely to develop leprosy (OR: 1.70; CI: 1.03-2.80; p < 0.05). Blood-related contacts (OR: 3.30; CI: 1.77-6.15; p < 0.001), member of household contacts (OR: 1.92; CI: 1.09 -3.40; p < 0.05),and age of contacts are statistically significantly more likely to develop leprosy at higher age groups 30–44 years (OR: 2.82; CI: 1.49-5.35; p < 0.05) and 45+ years (OR: 2.62; CI: 1.42-4.84; p < 0.05). Contacts associated with higher age of index patients are statistically significantly less likely to develop leprosy (OR:0.98; CI:0.97-0.99; p < 0.05). In year 2017, leprosy incidence was statistically significantly lower as compared to the contact enrolment year of 2012.

**Table 5:** Results of multilevel mixed-effects logistic regression analysis, Maltalep trial and non-intervention cohort.

Variable	Maltalep trial vs non-intervention cohort (n = 18,687)		
	Adjusted odds ratios (AOR)	Confidence Intervals (CIs)	p-values
**Intervention arm**			
Maltalep trial	1		
Non-intervention cohort	1.70	1.03-2.80	0.04
**Age of contacts (years)**			
5-14	1		
15-29	1.51	0.85-2.65	0.16
30-44	2.82	1.45-5.36	0.00
45+	2.62	1.42-4.84	0.00
**Genetic distance to index patients**			
Not blood related (or unknown)	1		
Blood-related (brother/sister/child/parent)	3.30	1.77-6.15	0.00
Blood-related (other)	1.57	0.91-2.73	0.10
**Physical distance to index patients**			
Not a household member	1		
Household member (sharing roof and kitchen)	1.92	1.09-3.40	0.02
**Age of index patient (years)**	0.98	0.97-0.99	0.02
**Leprosy classification index patient**			
PB	1		
MB	1.37	1.06-1.77	0.02
**Occupation index patient**			
Others	1		
Daily labor	1.44	0.76-2.73	0.26
Time point of Index patients’ enrolment (year)			
2012	1		
2013	0.36	0.12-1.11	0.08
2014	0.42	0.14-1.26	0.12
2015	0.42	0.14-1.29	0.13
2016	0.38	0.12-1.19	0.10
2017	0.18	0.04-0.84	0.03

BCG scar of index patient was not available in the dataset of non-intervention cohort, thus not included in the analysis. Analysis was adjusted for gender of both contacts and index patient.

## Discussion

The Maltalep trial assessed whether single-dose rifampicin (SDR) given after bacillus Calmette–Guérin (BCG) vaccination was able to prevent possible excess leprosy cases in contacts of newly diagnosed leprosy patients. The primary outcome measure of the trial was the incidence rate of leprosy per 10,000 population at risk among contacts during a 2-year observation period. For the current study we extended the observation period to 5 years. During these 5 years, there was no statistically significant difference in the incidence of leprosy between the contacts of the two arms of the trial. In comparing the Maltalep trial (both arms taken together) with the non-intervention cohort adjusted for observed factors, the odds ratio of the incidence was statistically significantly 1.70 times higher in the contacts of the non-intervention cohort. After adjusting for observed covariates in multilevel analysis, the total unobserved variation in leprosy outcome was 41% for the differences between index patients, and 60% for the differences between contacts within the same index patient group.

Regarding observed risk factors, SDR- arm contacts of MB, SSS positive, blood-related (brother/sister, child, parent), and ‘blood-related other’ to index patients had higher risks for leprosy compared to PB, SSS negative, and not blood-related index patients. However, contacts of MB and SSS positive index patients, and contacts of ‘blood-related other’ to index patients in the intervention arm (SDR+) were statistically significantly less likely to develop leprosy than contacts of MB, SSS positive index patients and contacts of ‘blood-related other’ in the SDR- arm (see also S4 Table). In multilevel analysis the contribution of genetic and physical distance to the index patients to the incidence of leprosy in the contacts was higher than in logistic regression analysis. Contacts with higher age (45+ years) were at increased risk of leprosy.

The original analysis of the two-year follow-up of the Maltalep trial established a 42% reduction of leprosy incidence in the first year after SDR was given. Although being a substantial reduction, it was not statistically significant at the p = 0.05 level [16]. Furthermore, there was no difference at all in the second year after SDR. The outcome after five years of observation confirms the doubtful additional benefit of SDR given to contacts soon after BCG vaccination to reduce excess leprosy among these contacts due to the vaccine. We had previously reported that these excess cases occur in the Maltalep trial very soon (within 8–12 weeks) after BCG vaccination [11]. In hindsight, SDR should possibly be given at least one week before BCG vaccination to sort a better protective effect. The sequence of these two interventions (first BCG and then SDR) was chosen because of logistic reasons in view of potential refusal of the contacts for the BCG vaccine during a second visit by the intervention team to their home. If SDR was given first and afterwards BCG there could have been less new cases (of the PB type) in both arms that became clinically evident within 12 weeks after BCG vaccination, but the immunological mechanism determining such phenomenon is as yet not fully understood and needs further study to decide on a possible beneficial combination of BCG with SDR.

The statistically significant difference in leprosy incidence between the intervention trial and non-intervention cohort (odds ratio 1.70) confirms the preventive effect of BCG vaccination against leprosy (regardless the addition of SDR). Although the non-intervention cohort was a separate study, the participants were recruited in the same project area and during the same period of intake into the Maltalep trial. The basic demographic characteristics of the index cases are comparable in both studies and adjusted statistically for differences in both arms of the Maltalep trial and the non-intervention cohort. Furthermore, the analysis was adjusted for the time of enrolment of contacts to account for the time variation in leprosy incidence. We therefore assume that there is value in comparing the two studies, but with due caution regarding the methodological limitation of this approach. The protective effect of BCG against leprosy in contacts is comparable with previous studies [10,12,14].

Taking advantage of the wealth of data gathered at the RHP in Bangladesh, we performed an in-depth analysis of the relationship between leprosy incidence and associated risk factors (covariates). In general, the covariate effects found in our current study are in line with existing studies [8,22]. However, the effects of the trial are explained largely by the differential risk factors and unobserved heterogeneity characteristics of the contacts in the trial. Unobserved heterogeneity factors influence the effects of covariates such as the age of contacts and relationship and distance of contact to the index case. The effect sizes were large in our multilevel analysis compared to the traditional logistic regression results, indicating the importance of addressing unobserved variations in the leprosy outcome at different levels of data [27]. Failing to include potential observed covariates in the regression analysis might lead to an unobserved heterogeneity (unobserved variability) in the outcome variable.

Further exploration of significant covariates of genetic and physical distance showed that 22% of all contacts of the Maltalep trial were ‘blood-related’ (first degree relatives), and 30% ‘blood-related other’ (second degree relatives). Of the ‘blood-related’ contacts, one quarter lived in the same household of index patients; this was the case for only 2% of the ‘blood-related other’ contacts. Thus, when comparing the effects of genetic and physical distance, we found a large and strong association between inherited susceptibility in terms of being a blood-related contact (first and second degree; 4.34 and 3.07 times higher odds, respectively) and the incidence of leprosy than the previously reported odds ratio 1.65 being a blood-related contact [22]. For understanding of transmission dynamics, it is necessary to disentangle the effect of blood-relationship (genetic distance) from the effect of living together closely (physical distance). Moet *et al.* reported in 2006 that both genetic and physical distance were independent predictors of developing leprosy, but their individual effects were not disentangled [22]. Furthermore, a recent study in Bangladesh showed no evidence of blood-relationship and leprosy incidence in their contacts [28].

Our robust analytical method of multilevel analysis addressing the clustering effects at the levels of new leprosy patients on the one hand and contacts of the new patients on the other hand, helped to determine and quantify more profoundly the effects of blood-relationship among contacts on the likelihood of leprosy incidence. The factors that drive these statistically significant differences in covariate effects remain unknown. We refer to them as biological, behavioral factors (e.g., how frequently contacts visit index patients), lack of seeking healthcare, poverty, and possibly including many other social factors. For instance, shortage of household food and lack of dietary diversity have been associated with the risk of leprosy [29,30]. In our analysis however, we could not control for indicators of poverty, dietary diversity, behavior, or other social and biological factors. Thus, they remain unobserved, but carry the risk of leprosy in contacts and enhance the effect of genetic factors through such unobserved pathways.

In line with existing studies, we did not find any statistically significant association between gender and leprosy incidence [8,31,32]. The higher the age of contacts, the higher the risk of developing leprosy. This was highest in the 45+ years age group.

In studying overall covariate effects, we confirmed the protective effect of BCG + SDR (SDR+) in the contacts of MB, SSS-positive, and ‘blood-related other’ index cases. The finding of the larger protective effect in contacts of SSS-positive index patients is new and interesting. Being a contact of an index patient with a positive SSS is known to be a dominant risk factor for leprosy [28]. This increased risk is also confirmed in our study, but we were also able to show that the contacts of index patients with a positive SSS benefit relatively more from BCG vaccination followed by SDR than contacts of SSS negative index patients.

In the Maltalep trial, index patients were assigned randomly into two arms. Random allocation ensures that treatment status is not confounded by either measured or unmeasured baseline characteristics. Thus, one can directly estimate the average treatment effect by subtracting the estimated outcomes between two treatment groups [33]. However, as the randomization was not done at contact (individual) level, it is possible that the contacts in the treatment groups differ in baseline observed and unobserved characteristics. Contacts in both arms of the Maltalep trial differed with regard to the covariates blood-related (first degree), blood-related other (second degree), physical distance (whether contacts are a household member or not), and index patient with PB/MB type, SSS-positive, and occupation of index patient (Table 2). The difference is small, but because large numbers of contacts were followed-up, and because it concerns an infectious disease, a small difference in characteristics of the contacts and their behavior (which we do not observe) may make a difference in the incidence rate of leprosy between the two study arms. It is possible that contact groups differ in unobserved behavioral, biological, and environmental factors. Therefore, it remains important to account for these differences when estimating the effect of an intervention on the leprosy incidence in the contacts of new patients.

Conditioning on the propensity score allows obtaining unbiased estimates of average treatment effects [34], while the independence of treatment assignment is strongly ignored. Existing studies lack adjustment for the variations at the levels of contacts while contacts are not randomized, and residual differences in baseline characteristics between two groups, which may limit the conclusions on treatment and covariates effects.

### Strengths and limitations of the study

The strengths of this study are: 1) the large and prospectively collected dataset with limited loss to follow-up, 2) the use of the IPW method in balancing the baseline characteristics between the two arms of the trial; and 3) the application of that weight in multilevel mixed effects logistic regression analysis, which helps to converge the model faster and obtain the cluster adjusted risk factor effects.

An important limitation of our current analysis is that data from two studies are compared that initially were not designed for such comparison. While the Maltalep study is a randomized-controlled trial with two intervention arms, the IDEAL study is a non-intervention cohort of patients and their contacts who were recruited separately from the trial. Both studies however, were conducted simultaneously in the same geographical project area, among the same population, and applying the same guidelines for case finding, diagnosis, and contact survey. We therefore, with due consideration to this methodological limitation and with careful statistical adjustment, have treated the non-intervention cohort as pseudo ‘control’ group for the Maltalep trial. Nevertheless, this limitation does reflect on the robustness of the outcomes when data of both studies are brought together and compared.

Another important consideration is loss to follow-up. In the Maltalep trial, less than 1% of all contacts were lost to follow-up completely over the five-year observation period. In the non-intervention cohort no contacts were lost to follow-up completely over the 5-year observation period. Such low loss to follow-up will only minimally influence the results of our analyses. While the follow-up of contacts in the Maltalep trial was yearly, the first follow-up of the non-intervention cohort started two years after enrollment of an index case. This 2-year delay could mean that some leprosy cases may have been missed or self-healed in the meantime compared to the Maltalep trial with its first follow-up after 1 year. This difference could potentially affect the observed incidence of leprosy, with an underestimation of the number of new cases in the non-intervention cohort and thus an underestimation of the effect of BCG vaccination.

## Conclusions and recommendations

BCG vaccination of contacts is effective in preventing leprosy, but there is no clear benefit in adding SDR after BCG to reduce the number of excess leprosy cases soon after vaccination. SDR after BCG, however, is effective to prevent leprosy in contacts of MB patients, smear positive index patients, and contacts of index patients that are blood-related in the second degree (e.g., cousins, etc.). Genetic relationship is a more profound risk factor for leprosy in contacts than being a household contact only. Ignoring taking into account for the leprosy clustering caused by unmeasured factors may bias the effect of risk factors, such as the age of contacts and genetic relationship of contact to the index case. Therefore, adjustment is needed for this in the outcome analysis.

## Supporting information

S1 Table Protective efficacy of Maltalep trial (SDR + arm versus SDR- arm) and non-intervention cohort in contacts of newly diagnosed leprosy patients over the pre-baseline and five-year observation period (FU1-FU5). (DOCX)

S2 Table PB leprosy in contacts of newly diagnosed leprosy (index) patients by study groups. (DOCX)

S3 Table MB leprosy in contacts of newly diagnosed leprosy (index) patients by study groups. (DOCX)

S4 Table Protective efficacy of BCG versus BCG and SDR prophylaxis in contacts of newly diagnosed leprosy patients by variable category at two years follow-up (FU2). (DOCX)

S5 Table Protective efficacy of BCG versus BCG and SDR prophylaxis in contacts of newly diagnosed leprosy patients by variable category at three years follow-up (FU3). (DOCX)

S6 Table Protective efficacy of BCG versus BCG and SDR prophylaxis in contacts of newly diagnosed leprosy patients by variable category at four years follow-up (FU4). (DOCX)

S7 Table Protective efficacy of BCG versus BCG and SDR prophylaxis in contacts of newly diagnosed leprosy patients by variable category at five years follow-up (FU5). (DOCX)

S8 Table Logistic regression results without using IPW. (DOCX)

S9 Table Results of logistic regression analysis, Maltalep trial SDR- and SDR+ arms, separately. (DOCX)

S10 Table Multilevel analysis of potential risk factors and leprosy incidence in the contacts of the Maltalep trial after SDR was given, N = 14,542. (DOCX)
