# Supplementary material for: Risk factors and leprosy incidence among contacts in Bangladesh: A multilevel analysis
Source: PLoS Negl Trop Dis. 2025 Sep 5;19(9):e0013465. doi: 10.1371/journal.pntd.0013465 (PMC12412996; doi:10.1371/journal.pntd.0013465)
Supplement: S1 Table — (DOCX) [file pntd.0013465.s001.docx]

**S1 Table. Protective efficacy of Maltalep trial (SDR+ arm versus SDR- arm) and non-intervention cohort in contacts of newly diagnosed leprosy patients over the pre-baseline and five-year observation period (FU1-FU5).**

| **Follow-ups** | **Leprosy % (n)** | **Odds Ratios (95% CI)** | **Pearson Chi2 (p-value)** |
| --- | --- | --- | --- |
| **Pre-baseline** |  |  |  |
| SDR+ arm, n=7,477 | 0.41 (31) | 1 | 0.69 (0.41) |
| SDR- arm, n=7,246 | 0.33 (24) | 0.80 [0.47, 1.46] |  |
| Non-intervention cohort | na | na |  |
| **FU1 (1 year)** |  | | |
| SDR+ arm, n=7,045 | 0.27 (19) | 1 | 1.54 (0.21) |
| SDR- arm, n=6,920 | 0.39 (27) | 1.47 [0.82,2.65] |  |
| Non-intervention cohort | na | na |  |
| **FU2 (2 years)** |  | | |
| SDR+ arm, n=6,894 | 0.44 (30) | 1 | 6.74 (0.03) |
| SDR- arm, n=6,768 | 0.35 (24) |  |  |
| Non-intervention cohort, n=4,008 | 0.70 (28) | 1.34 [1.05, 1.67] |  |
| **FU3 (3 years)** |  | | |
| SDR+ arm, n=6,855 | 0.22 (15) | 1 | 12.80(0.00) |
| SDR- arm, n=6,733 | 0.21 (14) |  |  |
| Non-intervention cohort, n=3,904 | 0.56 (22) | 1.63 [1.23, 2.45] |  |
| **FU4 (4 years)** |  | | |
| SDR+ arm, n=6,770 | 0.25 (17) | 1 | 5.72 (0.02) |
| SDR- arm, n=6,651 | 0.18 (12) |  |  |
| Non-intervention cohort, n=3,854 | 0.44 (17) | 1.43 [1.06, 1.93] |  |
| **FU5 (5 years)** |  |  |  |
| SDR+ arm, n=6,598 | 0.29 (19) | 1 | 1.35 (0.25) |
| SDR- arm, n=6,541 | 0.28 (18) |  |  |
| Non-intervention cohort, n=3,743 | 0.40 (15) | 1.19[0.88, 1.6] |  |

- CI 95% confidence interval, na = not available
