# Supplementary material for: Risk factors and leprosy incidence among contacts in Bangladesh: A multilevel analysis
Source: PLoS Negl Trop Dis. 2025 Sep 5;19(9):e0013465. doi: 10.1371/journal.pntd.0013465 (PMC12412996; doi:10.1371/journal.pntd.0013465)
Supplement: S2 Table — (DOCX) [file pntd.0013465.s002.docx]

**S2 Table. PB leprosy in contacts of newly diagnosed leprosy (index) patients by study groups.**

| **Year-Follow-ups (FU1-5) after BCG** | **Maltalep Trial, SDR-** | | | **Maltalep Trial, SDR+** | | | **Non-intervention cohort** | | |
| --- | --- | --- | --- | --- | --- | --- | --- | --- | --- |
|  | Leprosy | Incidence rate per 10,000 population | Number at risk | leprosy | Incidence rate per 10,000 population | Number at risk | leprosy | Incidence rate per 10,000 population | Number at risk |
| Baseline | - | - | 7,222 | - | - | 7,325 | - | - | - |
| 1 year FU 1 | 23 | 33 [95% CI: 20-47] | 6,920 | 14 | 20 [95% CI: 9-30] | 7,045 | - | - | - |
| 2-year FU 2 | 23 | 34 [95% CI: 20-48] | 6,768 | 25 | 36 [95% CI:22-51] | 6,894 | 24 | 63 [95%CI: 38-87] | 4,008 |
| 3-year FU 3 | 12 | 18 [95% CI: 8-28] | 6,733 | 14 | 20 [95% CI:10-31] | 6,855 | 17 | 44 [95% CI: 23-64] | 3,904 |
| 4-year FU 4 | 11 | 17 [95% CI: 7-26] | 6,651 | 15 | 22 [95% CI: 11-33] | 6,770 | 16 | 44 [95% CI: 23-65] | 3,854 |
| 5-year FU 5 | 16 | 24 [95% CI: 13-36] | 6,541 | 16 | 24 [95% CI: 12-36] | 6,598 | 15 | 45 [95% CI:24-67] | 3,744 |
| **Total leprosy (FU1-5)** | 85 |  |  | 84 |  |  | 72 |  |  |

- Note: Numbers at risk are based on Figure 1 & 2. Non-intervention cohort, FU1 was not done, FU2 starts after 2 years of contacts enrolment.
