# Supplementary material for: Risk factors and leprosy incidence among contacts in Bangladesh: A multilevel analysis
Source: PLoS Negl Trop Dis. 2025 Sep 5;19(9):e0013465. doi: 10.1371/journal.pntd.0013465 (PMC12412996; doi:10.1371/journal.pntd.0013465)
Supplement: S3 Table — (DOCX) [file pntd.0013465.s003.docx]

**S3 Table. MB leprosy in contacts of newly diagnosed leprosy (index) patients by study groups.**

| **Year-Follow-ups (FU1-5) after BCG** | **Maltalep Trial, BCG only** | | | **Maltalep Trial, BCG+SDR** | | | **Non-intervention cohort** | | |
| --- | --- | --- | --- | --- | --- | --- | --- | --- | --- |
|  | Leprosy | Incidence rate per 10,000 population | Number at risk | leprosy | Incidence rate per 10,000 population | Number at risk | leprosy | Incidence rate per 10,000 population | Number at risk |
| Baseline | - | - | 7,222 | - | 1.4 [95% CI: - 1.3-4.0] | 7,325 | - | - | - |
| 1- year FU 1 | 4 | 6.0 [95% CI: -0.1-12.0] | 6,920 | 5 | 7.2 [95% CI: 0.9-13.0] | 7,045 | - | - | - |
| 2-year FU 2 | 1 | 1.5 [95% CI: -1.4-4.3] | 6,768 | 5 | 7.3 [95% CI: 0.9-14.0] | 6,894 | 4 | 10.0 {95%CI: 1.6-24.0] | 4,008 |
| 3-year FU 3 | 2 | 3.0 [95%CI: -1.2-7.0] | 6,733 | 1 | 1.5 [95%CI: -1.4-4.3] | 6,855 | 5 | 13.0 [95% CI:1.6-24.4] | 3,904 |
| 4-year FU 4 | 1 | 1.5 [95% CI: -.1.5-4.5] | 6,651 | 2 | 3 [95% CI: -1.1-7.1] | 6,770 | 1 | 3.0 [95% CI: 2.0-13.0] | 3,854 |
| 5-year FU 5 | 2 | 3.1 [95% CI: -0.1-7.3] | 6,541 | 3 | 5 [95% CI: - 0.6-10.0] | 6,598 | 0 | - | 3,744 |
| **Total leprosy (FU1-5)** | 10 |  |  | 16 |  |  | 10 |  |  |

- Note: Numbers at risk are based on Figure 1 & 2. Non-intervention cohort, FU1 was not done, FU2 starts after 2 years of contacts enrolment.
