# Supplementary material for: Risk factors and leprosy incidence among contacts in Bangladesh: A multilevel analysis
Source: PLoS Negl Trop Dis. 2025 Sep 5;19(9):e0013465. doi: 10.1371/journal.pntd.0013465 (PMC12412996; doi:10.1371/journal.pntd.0013465)
Supplement: S4 Table — (DOCX) [file pntd.0013465.s004.docx]

**S4 Table. Protective efficacy of BCG versus BCG and SDR prophylaxis in contacts of newly diagnosed leprosy patients by variable category at two years follow-up (FU2).**

| **Variables** | Maltalep trial, n=14,986 | | Combined dataset, n=19,202 | |
| --- | --- | --- | --- | --- |
|  | **SDR- vs. SDR+** | | **Maltalep vs. Non-intervention cohort** | |
|  | OR (95% CI) * | p-value | OR (95% CI) * | p-value |
| **Age contacts (year)** |  |  |  |  |
| 5-14 | 0.58 (0.21,1.60) | 0.29 | 1.65 (0.64, 4.23) | 0.30 |
| 15-29 | 1.79 (0.60, 5.34) | 0.30 | 1.08 (0.39, 3.01) | 0.88 |
| 30-44 | 4.48 (0.97, 20.78) | 0.06 | 1.79 (0.66, 4.85) | 0.25 |
| >=45 | 0.84 (0.28, 2.51) | 0.76 | 2.61 (1.17, 5.85) | 0.02* |
| **Gender of contacts** |  |  |  |  |
| Male | 1.24 (0.58, 2.66) | 0.57 | 0.99 (0.45, 2.17) | 0.98 |
| Female | 1.22 (0.57, 2.60) | 0.62 | 2.60 (1.46, 4.65) | 0.00** |
| **Genetic distance** |  |  |  |  |
| Blood-related (brother/sister, child, parent) | 1.40 (0.56, 3.49) | 0.47 | 1.92 (0.94, 3.89) | 0.07 |
| Blood-related (other) | 0.74 (0.29, 1.87) | 0.52 | 1.03 (0.38, 2.77) | 0.96 |
| Not-blood related | 1.84 (0.68, 4.99) | 0.23 | 2.14 (0.98, 4.67) | 0.06 |
| **Physical distance** |  |  |  |  |
| Household member | 0.64 (0.15, 2.67) | 0.54 | 3.72 (1.46, 9.45) | 0.01* |
| Not a household member | 1.38 (0.77, 2.48) | 0.28 | 1.38 (0.79, 2.376) | 0.25 |
| **BCG scar observed in contacts** |  |  | na |  |
| Present | 1.10 (0.55, 2.21) | 0.79 |  |  |
| Absent | 1.44 (0.61, 3.37) | 0.40 |  |  |
| **Type of leprosy index patient** |  |  |  |  |
| PB | 2.24 (1.11, 4.54) | 0.03* | 1.48 (0.80, 2.75) | 0.21 |
| MB | 0.33 (0.11,1.01) | 0.05* | 2.21 (1.08, 4.48) | 0.03* |

*Odds Ratio (with 95% confidence interval); reference category SDR- in column 1; reference category is Maltalep groups in column 3
