# Supplementary material for: Risk factors and leprosy incidence among contacts in Bangladesh: A multilevel analysis
Source: PLoS Negl Trop Dis. 2025 Sep 5;19(9):e0013465. doi: 10.1371/journal.pntd.0013465 (PMC12412996; doi:10.1371/journal.pntd.0013465)
Supplement: S5 Table — (DOCX) [file pntd.0013465.s005.docx]

**S5 Table. Protective efficacy of BCG versus BCG and SDR prophylaxis in contacts of newly diagnosed leprosy patients by variable category at three years follow-up (FU3).**

| **Variables** | Maltalep trial, n=14,986 | | Combined dataset, n=19,202 | |
| --- | --- | --- | --- | --- |
|  | **SDR- vs. SDR+** | | **Maltalep vs. Non-intervention cohort** | |
|  | OR (95% CI) * | p-value | OR (95% CI) * | p-value |
| **Age contacts (year)** |  |  |  |  |
| 5-14 | 0.97 (0.20, 4.81) | 0.96 | 3.68 (1.12, 12.09) | 0.03* |
| 15-29 | 0.80 (0.22, 2.99) | 0.74 | 1.38 (0.42, 4.49) | 0.59 |
| 30-44 | 1.99 (0.36,10.85) | 0.43 | 4.52 (1.57, 13.07) | 0.01* |
| >=45 | 0.96 (0.24, 3.86) | 0.96 | 1.96 (0.64, 6.01) | 0.24 |
| **Gender of contacts** |  |  |  |  |
| Male | 5.00 (1.09, 22.84) | 0.04* | 2.55 (1.07, 6.06) | 0.03* |
| Female | 0.40 (0.14, 1.15) | 0.09 | 2.73 (1.32, 5.63) | 0.01* |
| **Genetic distance** |  |  |  |  |
| Blood-related (brother/sister, child, parent) | 1.42 (0.45, 4.49) | 0.55 | 1.67 (0.65, 4.26) | 0.281 |
| Blood-related (other) | 0.74 (0.20, 2.74) | 0.65 | 2.09 (0.70, 6.25) | 0.19 |
| Not blood-related | 1.00 (0.25, 4.03) | 0.99 | 4.62 (1.82, 11.71) | 0.00** |
| **Physical distance** |  |  |  |  |
| Household member (share same kitchen and roof) | 0.84 (0.23, 3.16) | 0.47 | 2.00 (0.71, 5.65) | 0.19 |
| Not a household member | 1.18 (0.49, 2.89) | 0.36 | 2.87 (1.48, 5.54) | 0.00** |
| **BCG scar observed in contacts** |  |  | na |  |
| Present | 1.34 (0.54, 3.34) | 0.87 |  |  |
| Absent | 0.66 (0.19, 2.34) | 0.54 |  |  |
| **Type of leprosy index patient** |  |  |  |  |
| PB | 0.95 (0.39, 2.27) | 0.90 | 2.39 (1.17, 4.90) | 0.02* |
| MB | 1.34 (0.36, 4.98) | 0.67 | 3.04 (1.23, 7.49) | 0.02* |

*Odds Ratio (with 95% confidence interval); reference category SDR- in column 1; reference category is Maltalep groups in column 3
