# Supplementary material for: Risk factors and leprosy incidence among contacts in Bangladesh: A multilevel analysis
Source: PLoS Negl Trop Dis. 2025 Sep 5;19(9):e0013465. doi: 10.1371/journal.pntd.0013465 (PMC12412996; doi:10.1371/journal.pntd.0013465)
Supplement: S6 Table — (DOCX) [file pntd.0013465.s006.docx]

**S6 Table. Protective efficacy of BCG versus BCG and SDR prophylaxis in contacts of newly diagnosed leprosy patients by variable category at four years follow-up (FU4).**

| **Variables** | Maltalep trial, n=14,986 | | Combined dataset, n=19,202 | |
| --- | --- | --- | --- | --- |
|  | **SDR- vs. SDR+** | | **Maltalep vs. Non-intervention cohort** | |
|  | OR (95% CI) * | p-value | OR (95% CI) * | p-value |
| **Age contacts (year)** |  |  |  |  |
| 5-14 | 0.95 (0.19, 4.71) | 0.95 | 3.54 (0.95, 13.19) | 0.06 |
| 15-29 | 0.75 (0.17, 3.36) | 0.71 | 1.79 (0.52, 6.12) | 0.35 |
| 30-44 | 2.37 (0.61, 9.19) | 0.21 | 1.87 (0.62, 5.59) | 0.26 |
| >=45 | 0.98 (0.24, 3.92) | 0.98 | 1.54 (0.46, 5.11) | 0.48 |
| **Gender of contacts** |  |  |  |  |
| Male | 1.00 (0.40, 2.52) | 1.00 | 1.88 (0.87,4.08) | 0.11 |
| Female | 1.55 (0.51, 4.75) | 0.44 | 2.26 (0.87,5.84) | 0.09 |
| **Genetic distance** |  |  |  |  |
| Blood-related (brother/sister, child, parent) | 0.68 (0.19, 2.40) | 0.54 | 1.59 (0.53, 4.75) | 0.41 |
| Blood-related (other) | 0.69 (0.15, 3.07) | 0.62 | 2.70 (0.85, 8.51) | 0.09 |
| Not blood-related | 3.38 (0.93, 12.30) | 0.06 | 1.97 (0.78, 4.94) | 0.15 |
| **Physical distance** |  |  |  |  |
| Household member (share same kitchen and roof) | 0.54 (0.10, 2.94) | 0.47 | 0.59 (0.07, 5.06) | 0.63 |
| Not a household member | 1.45 (0.65, 3.24) | 0.36 | 2.39 (1.27, 4.49) | 0.00** |
| **BCG scar observed in contacts** |  |  | na |  |
| Present | 1.07 (0.45, 2.53) | 0.87 |  |  |
| Absent | 1.49 (0.42, 5.29) | 0.54 |  |  |
| **Type of leprosy index patient** |  |  |  |  |
| PB | 1.26 (0.53, 3.00) | 0.60 | 2.09 (0.97, 4.50) | 0.06 |
| MB | 1.07 (0.31, 3.71) | 0.91 | 0.190 (0.72,5.01) | 0.19 |

*Odds Ratio (with 95% confidence interval); reference category SDR- in column 1; reference category is Maltalep groups in column 3
