# Supplementary material for: Risk factors and leprosy incidence among contacts in Bangladesh: A multilevel analysis
Source: PLoS Negl Trop Dis. 2025 Sep 5;19(9):e0013465. doi: 10.1371/journal.pntd.0013465 (PMC12412996; doi:10.1371/journal.pntd.0013465)
Supplement: S7 Table — (DOCX) [file pntd.0013465.s007.docx]

**S7 Table. Protective efficacy of BCG versus BCG and SDR prophylaxis in contacts of newly diagnosed leprosy patients by variable category at five years follow-up (FU5).**

| **Variables** | Maltalep trial, n=14,986 | | Combined dataset, n=19,202 | |
| --- | --- | --- | --- | --- |
|  | **SDR- vs. SDR+** | | **Maltalep vs. Non-intervention cohort** | |
|  | OR (95% CI) * | p-value | OR (95% CI) * | p-value |
| **Age of contacts (year)** |  |  |  |  |
| 5-14 | 0.98 (0.14, 6.93) | 0.98 | 5.56 (1.49, 20.73) | 0.01* |
| 15-29 | 1.00 (0.20, 4.97) | 1.00 | 1.57 (0.39, 6.30) | 0.52 |
| 30-44 | 0.76 (0.26, 2.18) | 0.60 | 1.12 (0.36, 3.49) | 0.84 |
| >=45 | 1.12 (0.41, 3.11) | 0.82 | 0.62 (0.18, 2.14) | 0.45 |
| **Gender of contacts** |  |  |  |  |
| Male | 0.89 (0.34, 2.32) | 0.82 | 1.71 (0.73, 3.99) | 0.22 |
| Female | 0.98 (0.42, 2.26) | 0.96 | 1.19 (0.50, 2.80) | 0.69 |
| **Genetic distance** |  |  |  |  |
| Blood-related (brother/sister, child, parent) | 1.33 (0.49, 3.57) | 0.58 | 0.54 (0.16,1.86) | 0.33 |
| Blood-related (other) | 0.92 (0.30, 2.86) | 0.89 | 0.94 (0.26,3.32) | 0.92 |
| Not blood related | 0.81 (0.22, 3.03) | 0.76 | 3.70 (1.47, 9.33) | 0.01* |
| **Physical distance** |  |  |  |  |
| Household member (share same kitchen and roof) | 2.72 (0.53,14.07) | 0.23 | 0.86 (0.18, 4.15) | 0.85 |
| Not a household member | 0.76 (0.38, 1.53) | 0.44 | 1.56 (0.81, 2.98) | 0.18 |
| **BCG scar observed in contacts** |  |  | na |  |
| Present | 0.98 (0.34, 2.80) | 0.97 |  |  |
| Absent | 0.92 (0.42, 2.03) | 0.85 |  |  |
| **Type of leprosy index patient** |  |  |  |  |
| PB | 0.82 (0.38, 1.77) | 0.62 | 1.51 (0.70, 3.24) | 0.30 |
| MB | 1.25 (0.42, 3.72) | 0.69 | 1.25 (0.48, 3.30) | 0.65 |

*Odds Ratio (with 95% confidence interval); reference category SDR- in column 1; reference category is Maltalep groups in column 3
