# Supplementary material for: Risk factors and leprosy incidence among contacts in Bangladesh: A multilevel analysis
Source: PLoS Negl Trop Dis. 2025 Sep 5;19(9):e0013465. doi: 10.1371/journal.pntd.0013465 (PMC12412996; doi:10.1371/journal.pntd.0013465)
Supplement: S8 Table — (DOCX) [file pntd.0013465.s008.docx]

**S8 Table. Logistic analysis of potential risk factors and leprosy incidence in the contacts of the Maltalep trial after SDR was given, N=14,547.**

| **Variables** | **Model** |  |
| --- | --- | --- |
| **Intervention** | AORs | p-value |
| SDR- | 1 |  |
| SDR+ | 1.08 (0.81-1.43) | 0.65 |
| **Age of contacts** |  |  |
| 5-14 | 1 |  |
| 15-29 | 1.65 (1.08-2.53) | 0.02 |
| 30-44 | 2.25 (1.46-3.46) | 0.00 |
| 44+ | 2.16 (1.41-3.31) | 0.00 |
| **Leprosy classification of index patients** |  |  |
| Paucibacillary (PB1-5) | 1 |  |
| Multibacillary (MB) | 1.40 (1.04-1.90) | 0.03 |
| **Genetic distance to index patients** |  |  |
| Not blood-related | 1 |  |
| Blood-related (Brother/sister, child, parent) | 2.41 (1.68-3.44) | 0.00 |
| Blood-related other | 1.56 (1.05-2.31) | 0.03 |
| **Physical distance to index patients** |  |  |
| Not a household member | 1 |  |
| Household member (KR) | 1.55 (1.07-2.26) | 0.02 |
| **Interaction with SDR+** |  |  |
| Multibacillary | - |  |
| Genetic distance (blood-related other) | - |  |
| **Sample size a** | 14,547 |  |
| **Used IPW** | no |  |
| **Loglikelihood** | -1003.88 |  |
| **Parameter** | 14 |  |
| **AIC** | 2035.76 |  |

**Note**

IPW Inverse probability weight. AIC Akaike Information Criteria; Adjusted risk factors for age of index patients, gender of both contacts and index patients, occupation of index patients as labor.
