# Supplementary material for: Risk factors and leprosy incidence among contacts in Bangladesh: A multilevel analysis
Source: PLoS Negl Trop Dis. 2025 Sep 5;19(9):e0013465. doi: 10.1371/journal.pntd.0013465 (PMC12412996; doi:10.1371/journal.pntd.0013465)
Supplement: S9 Table — (DOCX) [file pntd.0013465.s009.docx]

**S9 Table: Results of logistic regression analysis, Maltalep trial SDR- and SDR+ arms, separately.**

| **Variables** | **Maltalep trial SDR- arm, n=7,218**  **(AOR)** | **Confidence Intervals**  **(CIs)** | **Maltalep trial SDR+ arm, n=7,325**  **(AOR)** | **Confidence Intervals**  **(CIs)** |
| --- | --- | --- | --- | --- |
| **Age of contacts** |  |  |  |  |
| 5-14 | 1 | 1 | 1 | 1 |
| 15-29 | 1.80 | 1.00-3.22* | 1.52 | 0.82-2.82 |
| 30-44 | 1.73 | 0.91-3.28 | 2.79 | 1.54-5.01** |
| 45+ | 2.36 | 1.30-4.26** | 2.00 | 1.08-3.72* |
| **Gender of contacts** |  |  |  |  |
| Male | 1 | 1 | 1 | 1 |
| Female | 0.89 | 0.57-1.30 | 1.48 | 0.97-2.24 |
| **Genetic distance to index patients** |  |  |  |  |
| Not blood-related (or unknown) | 1 | 1 | 1 | 1 |
| Blood-related (brother/sister/child/parent) | 2.90 | 1.70-4.95*** | 1.98 | 1.22-3.22** |
| Blood-related (other) | 2.34 | 1.33-4.10** | 1.03 | 0.59-1.81 |
| **Physical distance to index patients** |  |  |  |  |
| Not a household member | 1 | 1 | 1 | 1 |
| Household member (sharing roof and kitchen) | 1.93 | 1.14-3.26* | 1.24 | 0.71-2.14 |
| **Leprosy classification index patient** |  |  |  |  |
| PB | 1 | 1 | 1 | 1 |
| MB | 1.99 | 1.30-3.06** | 1.01 | 0.65-1.56 |

*p<0.05; **p<0.01 ***p<0.001; Adjusted risk factors for age of index patients, gender of both contacts and index patients, occupation of index patients as labour
