# Supplementary material for: Risk factors and leprosy incidence among contacts in Bangladesh: A multilevel analysis
Source: PLoS Negl Trop Dis. 2025 Sep 5;19(9):e0013465. doi: 10.1371/journal.pntd.0013465 (PMC12412996; doi:10.1371/journal.pntd.0013465)
Supplement: S10 Table — (DOCX) [file pntd.0013465.s010.docx]

**S10 Table. Multilevel analysis of potential risk factors and leprosy incidence in the contacts of the Maltalep trial after SDR was given, N=14,547.**

| **Variables** | **Model 4** | **p-value** |
| --- | --- | --- |
| **Intervention** | AORs |  |
| SDR- | 1 |  |
| SDR+ | 1.24 (0.24-6.41) | 0.80 |
| **Age of contacts** |  |  |
| 5-14 | 1 |  |
| 15-29 | 2.45 (1.05-5.71) | 0.04 |
| 30-44 | 2.24 (0.90-5.56) | 0.08 |
| 45+ | 3.47 (1.42-8.51) | 0.01 |
| **Slit skin smear values of index patients** |  |  |
| Negative value | 1 |  |
| 1-6 values | 6.36 (2.42-16.72) | 0.00 |
| **Genetic distance to index patients** |  |  |
| Not blood-related | 1 |  |
| Blood-related (Brother/sister, child, parent) | 4.48 (1.84-10.90) | 0.00 |
| Blood-related other | 3.38 (1.35-7.97) | 0.01 |
| **Physical distance to index patients** |  |  |
| Not a household member | 1 |  |
| Household member (KR) | 2.65 (1.22-5.72) | 0.01 |
| **Leprosy suspects at contacts enrolment** |  |  |
| No | 1 |  |
| Yes | 1.78 (0.93-3.44) | 0.08 |
| **Time of contacts enrolment (year)** |  |  |
| 2012 | 1 |  |
| 2013 | 0.56 (0.23-1.40) | 0.22 |
| 2014 | 0.45 (0.18-1.16) | 0.10 |
| 2015 | 0.65 (0.26-1.63) | 0.36 |
| 2016 | 0.48 (0.18-1.28) | 0.14 |
| 2017 | 0.29 (0.05-1.49) | 0.14 |
| **Interaction with SDR+** |  |  |
| Smear 1-6 values | 0.18 (0.04-0.75) | 0.02 |
| Genetic distance (blood-related other) | 0.28 (0.08-0.95) | 0.04 |
| **Sample size a** | 14,542 |  |
| **Used IPW** | yes |  |
| **Loglikelihood** | -969.41 |  |
| **Parameter** | 31 |  |
| **AIC** | 2000.82 |  |
| **BIC** | 2235.81 |  |
| **ICC between index patients** | 0.41 |  |
| **ICC between contacts within same index patient** | 0.62 |  |

**Note**

ICC Intra-cluster correlation; IPW Inverse probability weight. AIC Akaike Information Criteria; BIC Bayesian information criterion; adjusted risk factors for age of index patients, gender of both contacts and index patients, occupation of index patients as labor and interaction terms.
